# Construction of a ferroptosis-related five-lncRNA signature for predicting prognosis and immune response in thyroid carcinoma

**DOI:** 10.1186/s12935-022-02674-z

**Published:** 2022-09-29

**Authors:** Yuan Qin, Dai Zhang, Huan Zhang, Lan Hou, Zhe Wang, Liu Yang, Mingkun Zhang, Ge Zhao, Qing Yao, Rui Ling, Juliang Zhang

**Affiliations:** Department of Thyroid, Breast, and Vascular Surgery, Xijing Hospital, The Air Force Medical University, Xi’an, People’s Republic of China

**Keywords:** Ferroptosis, lncRNA, Prognostic signature, Tumor immune microenvironment, Thyroid carcinoma

## Abstract

**Background:**

Thyroid carcinoma (THCA) is the most common endocrine-related malignant tumor. Despite the good prognosis, some THCA patients may deteriorate into more aggressive diseases, leading to poor survival. This may be alleviated by developing a novel model to predict the risk of THCA, including recurrence and survival. Ferroptosis is an iron-dependent, oxidative, non-apoptotic form of cell death initially described in mammalian cells, and plays an important role in various cancers. To explore the potential prognostic value of ferroptosis in THCA, ferroptosis-related long non-coding RNAs (FRLs) were used to construct model for risk prediction of THCA.

**Methods:**

RNA-sequencing data of THCA patients and ferroptosis-related genes were downloaded from The Cancer Genome Atlas (TCGA) and FerrDb, respectively. A total of 502 patients with complete data were randomly separated into a training cohort and a validation cohort at the ratio of 2:1. The Pearson correlation coefficients were calculated to determine the correlation between ferroptosis-related genes (FRGs) and the corresponding lncRNAs, and those meeting the screening conditions were defined as FRLs. Gene Expression Omnibus (GEO) database and qRT-PCR were used to verify the expression level of FRLs in THCA tissues. Univariate and multivariate cox regression analysis were performed to construct a FRLs signature based on lowest Akaike information criterion (AIC) value in the training cohort, then further tested in the validation cohort and the entire cohort. Gene set enrichment analysis (GSEA) and functional enrichment analysis were used to analyze the biological functions and signal pathways related to differentially expressed genes between the high-risk and low-risk groups. Finally, the relative abundance of different tumor-infiltrating immune cells were calculated by CIBERSORT algorithm.

**Results:**

The patients were divided into high-risk group and low-risk group based on a 5-FRLs signature (AC055720.2, DPP4-DT, AC012038.2, LINC02454 and LINC00900) in training cohort, validation cohort and entire cohort. Through Kaplan–Meier analysis and area under ROC curve (AUC) value, patients in the high-risk group exhibited worse prognosis than patients in the low-risk group. GEO database and qRT-PCR confirmed that LINC02454 and LINC00900 were up-regulated in THCA. Univariate and multivariate cox regression analyses showed that the risk score was an independent prognostic indicator. GSEA and functional enrichment analysis confirmed that immune-related pathways against cancer were significantly activated in the low-risk THCA patients. Further analysis showed that the immune cells such as plasma cells, T cells CD8 and macrophages M1, and the expression of immune checkpoint molecules, including PD-1, PD-L1, CTLA4, and LAG3, were remarkably higher in the low-risk group.

**Conclusion:**

Our study used the TCGA THCA dataset to construct a novel FRLs prognostic model which could precisely predict the prognosis of THCA patients. These FRLs potentially mediate anti-tumor immunity and serve as therapeutic targets for THCA, which provided the novel insight into treatment of THCA.

**Supplementary Information:**

The online version contains supplementary material available at 10.1186/s12935-022-02674-z.

## Introduction

Thyroid carcinoma (THCA) is the most common malignancy of human endocrine system. The latest follow-up prevalence study revealed that the incidence of THCA ranked fifth in female malignancies worldwide [1]. Papillary thyroid carcinoma (PTC) is the most common pathological subtype of THCA, accounting for 85% to 90% of the total incidences. Due to the relatively inert biological behavior of PTC, its overall prognosis is relatively good, and the 10-year survival rate of patients is greater than 90% [2]. However, many cases still die of THCA due to malignant pathological subtypes, postoperative recurrence and distant metastasis [3]. Therefore, finding out novel diagnostic and prognostic markers as well as new therapeutic targets for THCA is of great significance.

Ferroptosis is generally referred to as one type of regulated cell death involving the production of iron-dependent reactive oxygen species (ROS), which is distinct from other forms of cell death regarding the morphological, biochemical, and genetic features [4, 5]. As revealed by more and more studies, ferroptosis plays an important role in tumor progression and treatment [6, 7]. Besides, various tumor types, such as lung adenocarcinoma, hepatocellular carcinoma, and ovarian cancer, have been demonstrated to be sensitive to ferroptosis [8, 9]. Similarly, ferroptosis has been proved as an essential part in THCA. Recent research has found that circular RNA circ_0067934 could attenuate ferroptosis of THCA cells by miR-545-3p/SLC7A11 signaling [10]. Another study has reported that knockdown of ETV4 could inhibit the PTC development by promoting ferroptosis through downregulating SLC7A11 [11].

In recent years, immune checkpoint blockage therapy has increasingly attracted the attention of researchers due to its great breakthrough in cancer immunotherapy. Immune checkpoint inhibitors targeting programmed cell death protein 1 (PD-1), programmed cell death ligand 1 (PD-L1), T cell immunoglobulin and ITIM domain (TIGIT), T cell immunoglobulin mucin-3 (TIM-3), and cytotoxic T lymphocyte antigen 4 (CTLA4) have been effective in treatment of various cancer types [12]. The response to immune checkpoint blockage therapy is closely related to the tumor microenvironment (TME). Ferroptosis related damage may result in inflammation-induced immunosuppression in the TME, facilitating tumor development [13]. Surprisingly, a research showed that CD8 + T cells with anti-tumor activity promote ferroptosis by down-regulating SLC3A2 and SLC7A11 [13]. However, the in-depth mechanisms of the interaction between ferroptosis and TME are still unclear. Therefore, exploring the relationship between ferroptosis and TME can help us better understand the pathogenesis of THCA and promote the development of treatment strategies.

The long non-coding RNAs (lncRNAs) are defined as the RNAs with over 200 nucleotides in length and without protein-coding ability [14, 15]. Increasing studies have demonstrated that the abnormal expression of lncRNAs exhibits both tumor-supportive or tumor-suppressive effect in various cancers [16–18]. Recent studies have indicated that dysregulation of specific lncRNAs was inextricably linked with the ferroptosis of malignant tumors [19, 20]. It was reported that upregulation of lncRNA OIP5-AS1 inhibited ferroptosis in prostate cancer with long-term cadmium exposure through miR-128-3p/SLC7A11 signaling [21]. Another study revealed that upregulation of lncRNA LINC00618 promoted vincristine-induced ferroptosis in human leukemia [22]. However, the complete role of lncRNAs in ferroptosis process of THCA remains obscure. The prognostic value of ferroptosis-related lncRNAs (FRLs) for THCA patients has never been systematically evaluated.

In this study, we aimed to identify FRLs in THCA, and provide important insight on the biological significance of ferroptosis in THCA. Furthermore, we analyzed the relationship between FRLs and immune microenvironment in THCA. FRLs were found as both prognostic markers and potential therapeutic targets of THCA patients.

## Materials and methods

### Data acquisition

The Cancer Genome Atlas (TCGA), a database with tremendous amounts of genomic and clinical data, facilitates relevant researches for genetic alterations and pathways that influence tumorigenesis, tumor progression, tumor differentiation, and tumor metastasis [23]. The RNA-sequencing (RNA-seq) data of 58 adjacent non-tumorous tissues (N) and 502 tumor tissues (T) as well as the corresponding clinical information of 502 THCA patients (patients with incomplete follow-up data were excluded) were downloaded from TCGA database (https://portal.gdc.cancer.gov/repository). The lncRNAs and protein-coding genes were classified by the Ensembl human genome browser GRCh38. p13 (http://asia.ensembl.org/index.html) [24]. FRGs were obtained from an authoritative public database (241 FRGs were obtained and their detailed information is provided in Additional file 1: Table S1), FerrDb (http://www.zhounan.org/ferrdb/), which provides the information of markers, regulators, and inducers of ferroptosis [25]. The present study did not require approval from an ethics committee because TCGA and FerrDb are publicly accessible databases.

### Establishment and verification of the prognostic model

The Pearson correlation coefficients were calculated to determine the correlation between FRGs and the corresponding lncRNAs. The FRLs were identified with the p value less than 0.001 (p < 0.001) and the absolute value of Pearson correlation coefficient more than 0.3 (|R|> 0.3). After normalizing data from TCGA database, the “limma” R package was used to obtain differentially expressed lncRNAs between tumor tissues and non-tumorous tissues based on the criteria of false discovery rate (FDR) < 0.05 and |log2FC|≥ 1 [26]. A total of 502 patients were randomly separated into a training cohort and a validation cohort at the ratio of 2:1 for constructing and validating the FRLs signature. Univariate cox regression analysis was performed to identify prognostic lncRNAs regarding OS (p < 0.05) in the training cohort. The intersected lncRNAs of differentially expressed lncRNAs, FRLs and prognostic lncRNAs were identified as the candidate lncRNAs for developing the FRLs prognostic signature. Then, multivariate cox regression analysis was performed on the candidate FRLs to evaluate their prognostic value. We identified five optimal FRLs for constructing the prognostic model based on lowest Akaike information criterion (AIC) value. The risk score of each patient was calculated according to the normalized expression levels of FRLs and their corresponding regression coefficients. The computational formula was as follows: Risk Score = e^sum (corresponding regression coefficient × each lncRNA’s expression)^. Based on the median value of risk score, we divided the patient into high-risk and low-risk groups in the training cohort, validation cohort and entire cohort, respectively. Kaplan–Meier (KM) survival curves with log-rank tests were used to analyze differences in OS between high-risk and low-risk groups. Then, time-dependent ROC curve was generated with “survival ROC” R package to evaluate the predictive accuracy of the FRLs signature.

### Sample collection

Eight pairs of THCA tissues and corresponding adjacent non-cancerous tissues were obtained from patients undergoing thyroidectomy at the Xijing Hospital from 2018 to 2020. All samples were immediately dissected, placed on ice, snap-frozen in liquid nitrogen, then stored at − 80℃ until use. The patient tissue samples were confirmed by histopathological examination to be PTC tissues and adjacent non-cancerous tissues. None of the patients had received preoperative local or systemic treatment. All procedures involving human participants in the study were in accordance with the ethical standards of the Research Ethics Committee of The Air Force Medical University as well as the 1964 Helsinki declaration and its later amendments.

### Total RNA isolation and quantitative real-time PCR (qRT-PCR)

Total RNA was isolated from frozen tissue and cell samples by RNAiso (Takara, Dalian, China). A reverse transcription kit (RR036A, Takara, Shiga, Japan) was used to transcribe total RNA and produce complementary DNA. For the analysis of gene expression, qRT-PCR was performed using SYBR Premix Ex Taq II (Takara) and the LightCycler 480 system (Roche, Indianapolis, IN, USA). The relative expression levels were calculated using the 2^−ΔCt^ method (Ct, cycle threshold). ΔCt indicates the difference in the Ct value between a target gene and the endogenous reference. GAPDH was used as the internal control. Each PCR was performed in triplicate to verify the stability and repeatability of the results. The primer sequences are available in Additional file 3: Table S3.

### Construction of the lncRNA-mRNA co-expression network

In order to demonstrate the correlation between the FRLs and their corresponding FRGs, the lncRNA-mRNA co-expression network was constructed and visualized using the Cytoscape software (version 3.7.2, http://www.cytoscape.org/). Then, the Sankey diagram was plotted to further demonstrate the correlation degree between FRLs and their corresponding FRGs.

### Gene set enrichment analysis and functional enrichment analysis

The “edgeR” R package was used to identify the differentially expressed genes between the high-risk and low-risk groups with the criteria of FDR < 0.05 and |log2FC|≥ 1. The identified differentially expressed genes were analyzed by gene set enrichment analysis (GSEA; http://www.broadinstitute.org/gsea) to explore the molecular and biological differences between the two groups. The gene sets were filtered based on the minimum and maximum sizes of 10 and 500 genes, respectively. In addition, Gene Ontology (GO) enrichment analysis was performed to determine the biological processes, molecular functions, and cellular components related to the FRLs signature. And the Kyoto Encyclopedia of Genes and Genomes (KEGG) enrichment analysis was performed to identify the signaling pathways associated with the FRLs signature.

### Estimation of tumor-infiltrating immune cells

The relative abundance of different tumor-infiltrating immune cells were calculated by CIBERSORT algorithm [27]. The normalized gene expression data were uploaded to the CIBERSORT web portal (http://cibersort.stanford.edu/), and the algorithm was based on LM22 gene signature and 1,000 permutations. The samples were filtered based on a p value < 0.05.

### Statistical analysis

Wilcox-test was used to compare relative abundance of tumor-infiltrating immune cells and expression levels of immune checkpoint molecules between high-risk and low-risk groups. Spearman correlation analysis was used to analyze the correlation between tumor-infiltrating immune cells. The proportions of patients with different clinical characteristics between groups were analyzed by the Chi-squared test. Univariate Cox regression analysis and multivariate Cox regression analysis were performed to identify independent prognostic factors. The predictive accuracy of the prognostic model regarding OS was evaluated by time-dependent ROC curve. All statistical analyses were conducted by SPSS (Version 21.0) or R software (Version 3.5). Statistical significance was defined as a p value < 0.05, and all tests were two-tailed.

## Results

### The clinical characteristics of patients in the training cohort, validation cohort and entire cohort

**Fig. 1:**
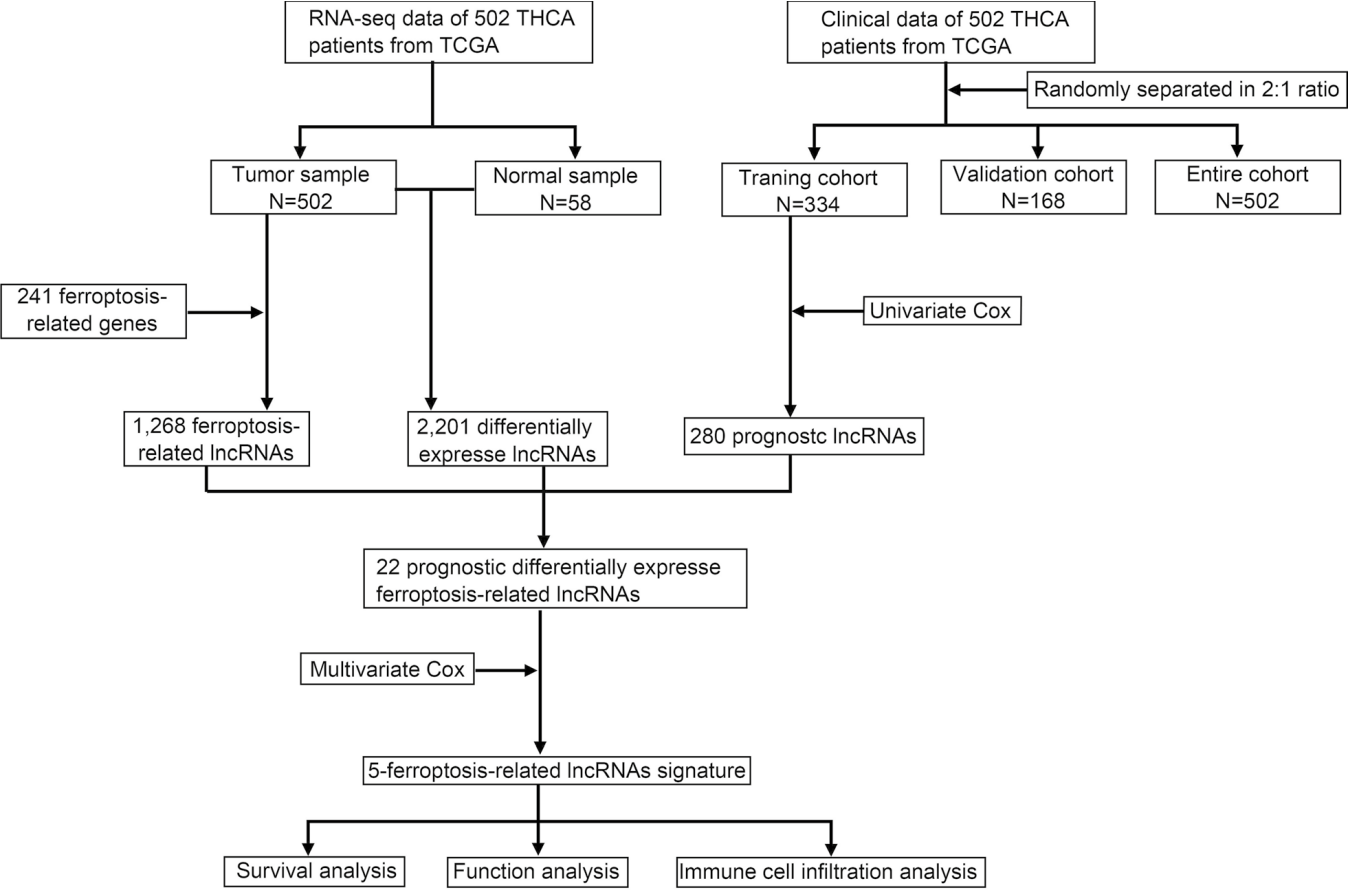
The flow diagram of this study

A total of 502 THCA patients from the TCGA database were defined as the entire cohort. They were randomly divided into a training cohort and a validation cohort at a ratio of 2:1 (n = 334 and 168, respectively). The detailed clinical characteristics of patients are presented in Table 1. There was no significant difference in clinical characteristics of patients between the training cohort and the validation cohort (Fig. 1).

**Table 1:** Clinical characteristics of patients in entire cohort, training cohort and validation cohort

Variables	Entire cohort (n = 502)		Training cohort (n = 334)		Validation cohort (n = 168)		*p*-value
	No	%	No	%	No	%	
Age							
Median(years)	46	–	46	–	46	–	–
< 55	335	66.7	224	67.1	111	66.1	0.823
≥ 55	167	33.3	110	32.9	57	33.9	
Gender							
Female	367	73.1	248	74.3	119	70.8	0.41
Male	135	26.9	86	25.7	49	29.2	
AJCC Stage							0.09
I	396	78.9	269	80.5	127	75.6	
II	84	16.7	46	13.8	38	22.6	
III	17	3.4	16	4.8	1	0.6	
IV	5	1	3	0.9	2	1.2	
T Stage							0.61
T1	145	28.9	94	28.1	51	30.4	
T2	164	32.7	111	33.2	53	31.5	
T3	170	33.9	111	33.2	59	35.1	
T4	23	4.6	18	5.4	5	3	
N Stage							0.207
N0	229	45.6	147	44	82	48.8	
N1	223	44.4	155	46.4	68	40.5	
Nx	50	10	32	9.6	18	10.7	
M Stage							0.471
M0	282	56.2	188	56.3	94	56	
M1	9	1.8	5	1.5	4	2.4	
Mx	211	42	141	42.2	70	41.6	

### Identification of prognostic differentially expressed FRLs in THCA patients

**Fig. 2:**
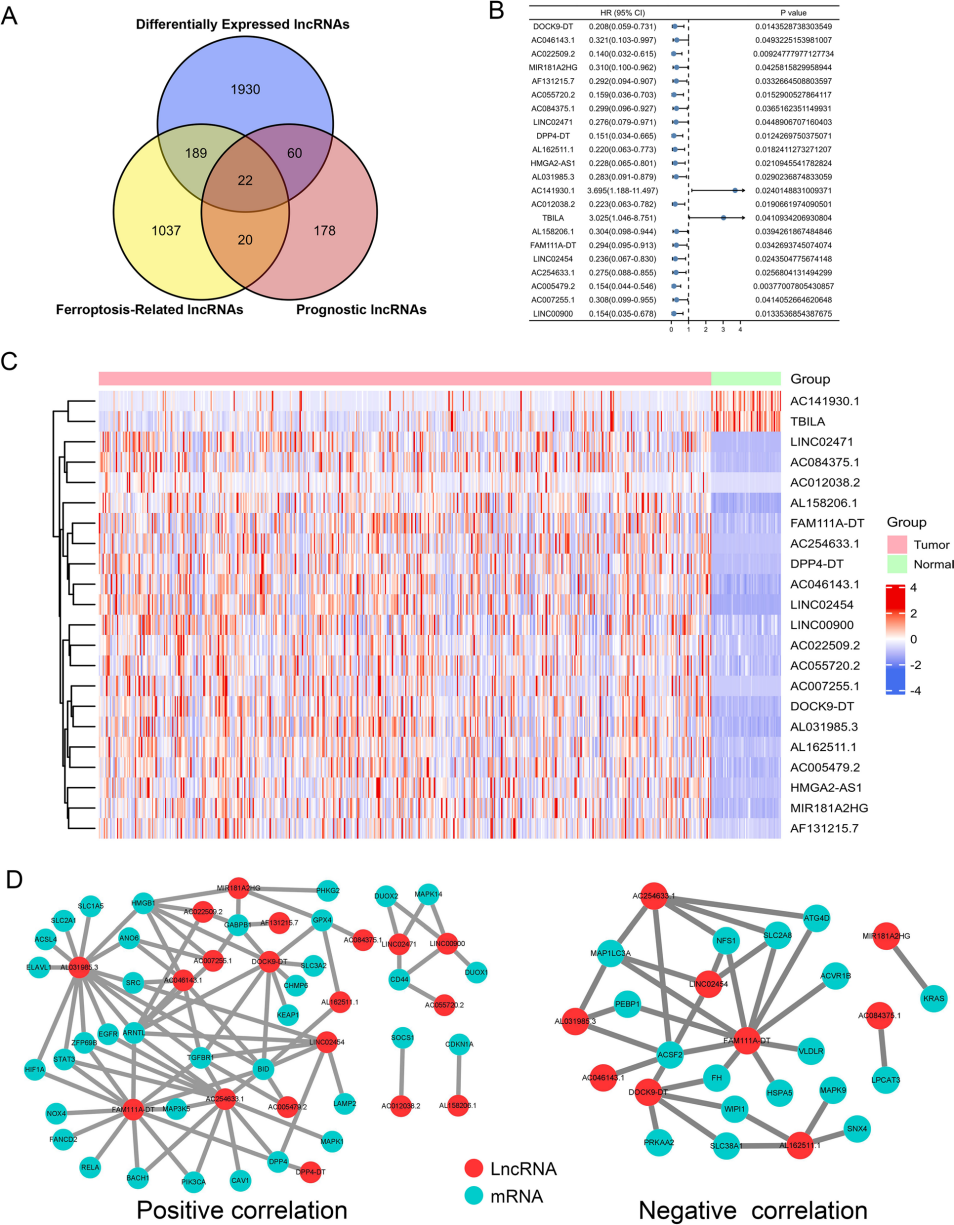
Identification of prognostic differentially expressed FRLs in THCA patients. **A** Venn diagram was established to identify the common lncRNAs of differentially expressed lncRNAs, FRLs, and prognostic lncRNAs. **B** The 22 intersected lncRNAs were differentially expressed in normal and tumor tissues. **C** Forest plot showing the univariate cox regression analysis on 22 lncRNAs. **D** lncRNA-mRNA co-expression network of candidate FRLs and FRGs. The left indicates positive correlation between FRLs and FRGs and the right indicates negative correlation between FRLs and FRGs

Firstly, a total of 502 THCA samples and 58 normal thyroid tissue samples were included for analyses. A total of 14,062 lncRNAs were identified by analyzing the RNA-seq data of the THCA patients in TCGA database. According to the threshold of adjusted p value < 0.05 and |log2 FC|≥ 1, 2,201 lncRNAs were found to be differentially expressed between tumor and normal tissues. Then, we identified 280 prognostic lncRNAs by univariate cox regression analysis (p < 0.05) in the training cohort. To identify FRLs, 241 ferroptosis-associated genes (FRGs) were downloaded from the ferroptosis database. We found that 1,268 FRLs were significantly correlated with FRGs (|R|> 0.3 and p < 0.001). Finally, Venn diagrams were used to exhibit the intersected lncRNAs of lncRNAs, prognostic lncRNAs and FRLs. We identified 22 lncRNAs (DOCK9-DT, AC046143.1, AC022509.2, MIR181A2HG, AF131215.7, AC055720.2, AC084375.1, LINC02471, DPP4-DT, AL162511.1, HMGA2-AS1, AL031985.3, AC141930.1, AC012038.2, TBILA, AL158206.1, FAM111A-DT, LINC02454, AC254633.1, AC005479.2, AC007255.1 and LINC00900) that were shared by three lncRNA sets, and these 22 lncRNAs were defined as prognostic differentially expressed FRLs between normal and tumor tissues (Fig. 2A–D).

### Construction of prognostic model based on frls in the training cohort

The expression levels of the 22 FRLs were used to construct a prognostic model by multivariate cox regression analysis in the training cohort regarding the overall survival (OS). An optimal 5-lncRNAs (AC055720.2, DPP4-DT, AC012038.2, LINC02454 and LINC00900) signature was identified based on the lowest Akaike information criterion (AIC) (Additional file 2: Table S2). The risk score was calculated using the following formula: e^(−0.347 × expression level of AC055720.2 − 1.923 × expression level of DPP4−DT − 0.591 × expression level of AC012038.2 + 0.43 × expression level of LINC02454 − 0.91 × expression level of LINC00900)^. The patients were further divided into a high-risk group (n = 167) and a low-risk group (n = 167) based on the median value of risk score. The risk score was significantly associated with T stage and N stage of THCA cancer patients (Table 2).

**Table 2:** Relationship between risk score and clinical characteristics of patients in the training cohort

Variables	Low-risk score (n = 167)		High-risk score (n = 167)		*p*-value
	No	%	No	%	
Age					
Median(years)	46	–	47	–	–
< 55	111	66.5	113	67.7	0.816
≥ 55	56	33.5	54	32.3	
Gender					
Female	124	74.3	124	74.3	1
Male	43	25.7	43	25.7	
AJCC Stage					0.49
I	130	77.8	139	83.2	
II	26	15.6	20	12	
III	10	6	6	3.6	
IV	1	0.6	2	1.2	
T Stage					0.034
T1	58	34.7	36	21.6	
T2	55	32.9	56	33.5	
T3	47	28.1	64	38.3	
T4	7	0.6	11	6.6	
N Stage					0.753
N0	75	44.9	72	43.1	
N1	78	46.7	77	46.1	
Nx	14	8.4	18	10.8	
M Stage					0.295
M0	87	52.1	101	60.5	
M1	3	1.8	2	1.2	
Mx	77	46.1	64	38.3	

**Fig. 3:**
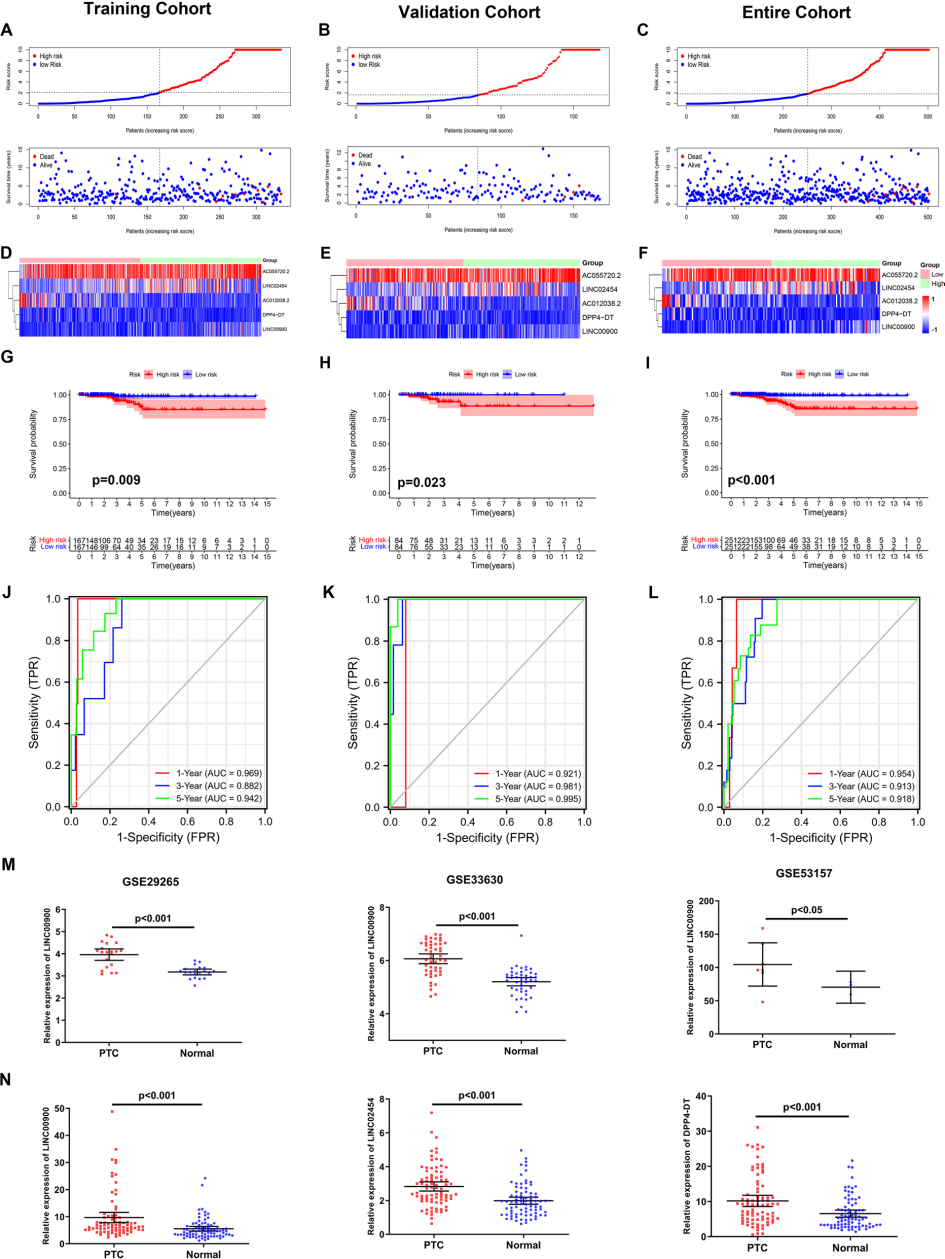
Prognostic analysis of the FRLs signature model in the training cohort, validation cohort and entire cohort. The distribution of the risk score and survival status in the **A** training cohort, **B** validation cohort and **C** entire cohort. Heatmap of five FRLs between the high-risk and low-risk groups in the **(D)** training cohort, **E** validation cohort and **F** entire cohort. Kaplan–Meier curves for the OS between the high-risk and low-risk groups in the **G** training cohort **H** validation cohort and **I** entire cohort. AUC of time-dependent ROC curves verified the prognostic accuracy of the risk score in the **J** training cohort, **K** validation cohort and **L** entire cohort. **M** LINC00900 expression in THCA tissues and normal tissues from GEO database. **N** The relative mRNA expression of LINC02454, LINC00900 and DPP4-DT in 80 paired THCA tissues and adjacent non-cancerous tissues

As shown in Fig. 3A, patients in the high-risk group presented decreased survival compared with patients in the low-risk group. And the Kaplan–Meier analysis showed that patients in the high-risk group had significantly worse OS than patients in the low-risk group (Fig. 3G, p = 0.009). The area under ROC curve (AUC) value reached 0.969 at 1 year, 0.882 at 3 years, and 0.962 at 5 years (Fig. 3J).

To test the reliability of the FRLs signature constructed in the training cohort, risk scores of the patients in the validation cohort and the entire cohort were calculated as described above. The patients in validation cohort were divided into a high-risk group (n = 84) and a low-risk group (n = 84), and the patients in entire cohort were also divided into a high-risk group (n = 251) and a low-risk group (n = 251) based on the corresponding median value of risk scores, respectively. Likewise, patients in the high-risk group of validation cohort and entire cohort were associated with worse survival outcome (Fig. 3B and 3C). And the Kaplan–Meier analysis showed that patients in the high-risk group had worse OS than patients in the low-risk group in both the validation cohort and the entire cohort (Fig. 3H and 3I, p = 0.023 and p < 0.001, respectively). The AUC values in the validation and entire cohorts reached 0.921 and 0.954 at 1 year, 0.981 and 0.913 at 2 years, and 0.995 and 0.918 at 5 years, respectively (Fig. 3K and L).

Then, we used three GEO databases (GSE29265, GSE33630, GSE53157) to verify the expression difference of FRLs between THCA and normal tissues. However, due to the limitation of sequencing platform, we can only obtain the expression of LINC00900. The results showed that the expression of LINC00900 in THCA was higher than that in normal tissues in all three GEO databases (Fig. 3M). Finally, qRT-PCR was used to detect the expression of LINC02454, LINC00900 and DPP4-DT in 80 pairs of THCA and paired adjacent tissues, and the expression of LINC02454, LINC00900 and DPP4-DT significantly increased in THCA (Fig. 3N).

### Independent prognostic value of the five-ferroptosis-related lncRNAs model

**Fig. 4:**
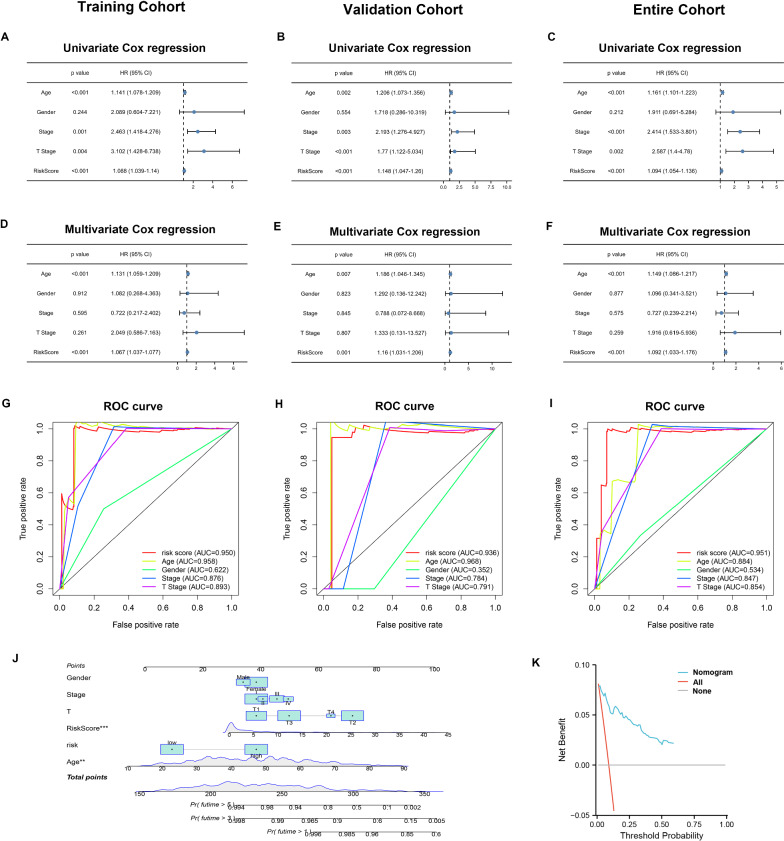
Independent prognostic value of the FRLs signature. Results of the univariate cox regression analysis and multivariate cox regression analysis regarding OS in the **A** and **D** training cohort, **B** and **E** validation cohort and **C** and **F** entire cohort. AUC of ROC curves compared the prognostic accuracy of the risk score and other prognostic factors in the **G** training cohort, **H** validation cohort and **I** entire cohort. **J** Nomogram to predict survival analysis for THCA patients. **K** Decision curve analysis (DCA) of the nomogram for predicting the overall survival (OS)

To determine whether the risk score was an independent prognostic factor for THCA patients, univariate cox regression and multivariate cox regression analyses were performed on the clinical characteristics and risk score. The results of univariate cox regression analysis showed that the risk score was significantly associated with OS in the training cohort, validation cohort and entire cohort (training cohort: HR = 1.088, 95% CI = 1.039–1.14, p < 0.001; validation cohort: HR = 1.148, 95% CI 1.047–1.26, p < 0.001; entire cohort: HR = 1.094, 95% CI 1.054–1.136, p < 0.001) (Fig. 4A–C). After adjusting for other confounders, the risk score remained to be an independent predictor of OS in the multivariate cox regression analysis (training cohort: HR = 1.067, 95% CI = 1.037–1.077, p < 0.001; validation cohort: HR = 1.16, 95% CI 1.031–1.206, p = 0.001; entire cohort: HR = 1.092, 95% CI 1.033–1.176, p < 0.001) (Fig. 4D–F).

Furthermore, the ROC curve showed that the AUC values of the FRLs prognostic signature in the training cohort, validation cohort and entire cohort were 0.95, 0.936 and 0.951, respectively, which were higher than the AUC values of other traditional prognostic factors (Fig. 4G–I). Based on the analysis results of multivariate logistic regression, the independent variables including age, gender, stage, T stage, and risk score were screened out for establishing a visualized nomogram to predict survival analysis for individual THCA patients (Fig. 4J). The decision curve analysis (DCA) showed that the prediction ability of the nomogram was more effective than a treat-none or treat-all strategy (Fig. 4K).

### Construction of the lncRNA-mRNA co-expression network

**Fig. 5:**
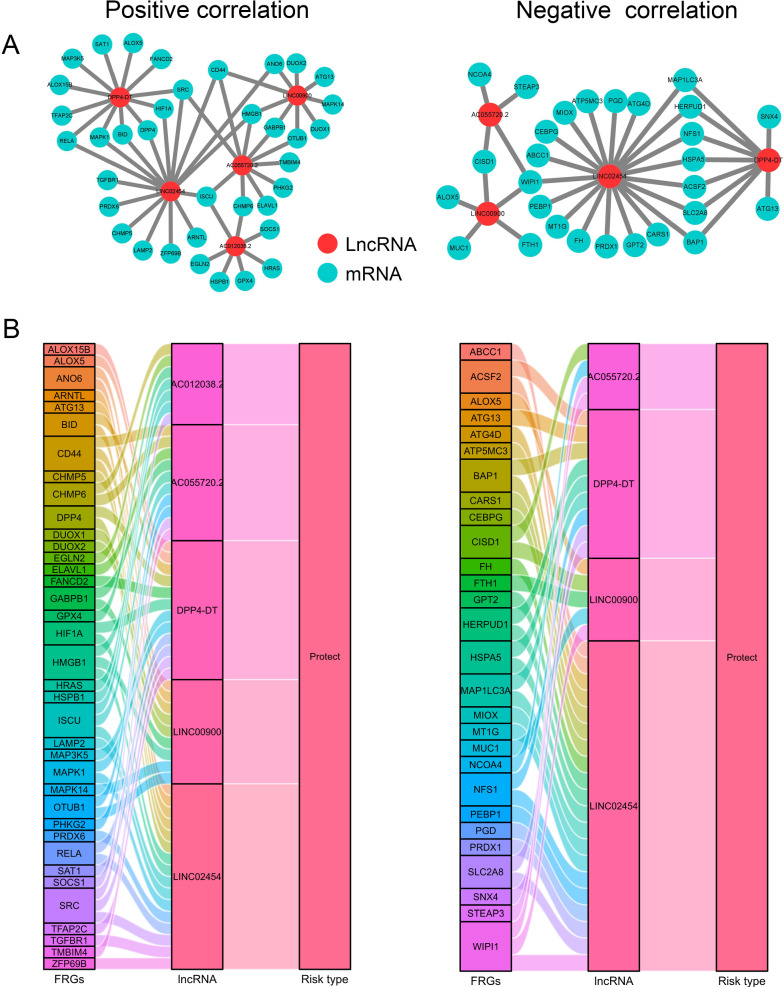
Construction of the FRLs–FRGs co-expression network **A** Diagram of the FRLs–FRGs network. The left indicates positive correlation between FRLs and FRGs and the right indicates negative correlation between FRLs and FRGs. **B** The Sankey diagram showing the connection degree between the FRLs and FRGs. The left indicates positive correlation between FRLs and FRGs and the right indicates negative correlation between FRLs and FRGs

To further explore the potential roles of FRLs in THCA, the lncRNA-mRNA co-expression network was constructed using Cytoscape for elucidating the correlation between FRLs and FRGs. The lncRNA-mRNA co-expression network included 92 pairs in total, among which 54 pairs were positively correlated and 38 pairs were negatively correlated (Fig. 5A). Within the network, LncRNA LINC02454 positively correlated with 16 FRGs (ARNTL, TGFBR1, LAMP2, HMGB1, CHMP5, ANO6, RELA, MAPK1, DPP4, BID, SRC, ISCU, PRDX6, ZFP69B, HIF1A, CD44) and negatively correlated with 20 FRGs (ABCC1, HSPA5, CARS1, PEBP1, GPT2, HERPUD1, MAP1LC3A, ACSF2, FH, ATG4D, NFS1, PRDX1, PGD, SLC2A8, ATP5MC3, WIPI1, MT1G, CEBPG, MIOX, BAP1). LncRNA DPP4-DT had positive relationship with 12 FRGs (SAT1, ALOX15B, MAP3K5, TFAP2C, FANCD2, RELA, MAPK1, DPP4, BID, SRC, ALOX5, HIF1A) and negative relationship with 9 FRGs (HSPA5, HERPUD1, MAP1LC3A, ACSF2, NFS1, SLC2A8, ATG13, BAP1, SNX4). Nine FRGs (OTUB1, HMGB1, GABPB1, ANO6, DUOX2, MAPK14, DUOX1, ATG13, CD44) positively correlated with lncRNA LINC00900 and 5 FRGs (MUC1, FTH1, ALOX5, WIPI1, CISD1) negatively correlated with lncRNA LINC00900, respectively. LncRNA AC055720.2 positively correlated with 10 FRGs (CHMP6, OTUB1, TMBIM4, PHKG2, HMGB1, GABPB1, SRC, ISCU, CD44 and ELAVL1) and negatively correlated with 4 FRGs (STEAP3, WIPI1, NCOA4, CISD1). Only lncRNA AC012038.2 was positively related to 7 FRGs (CHMP6, EGLN2, HRAS, GPX4, SOCS1, ISCU, HSPB1), with no negatively related FRGs being detected.

The Sankey diagram not only demonstrated the relationship between FRLs and FRGs, but also demonstrated the relationship between FRLs and OS of THCA patients (Fig. 5B).

### Explore cancer related pathways by gene set enrichment analysis

**Fig. 6:**
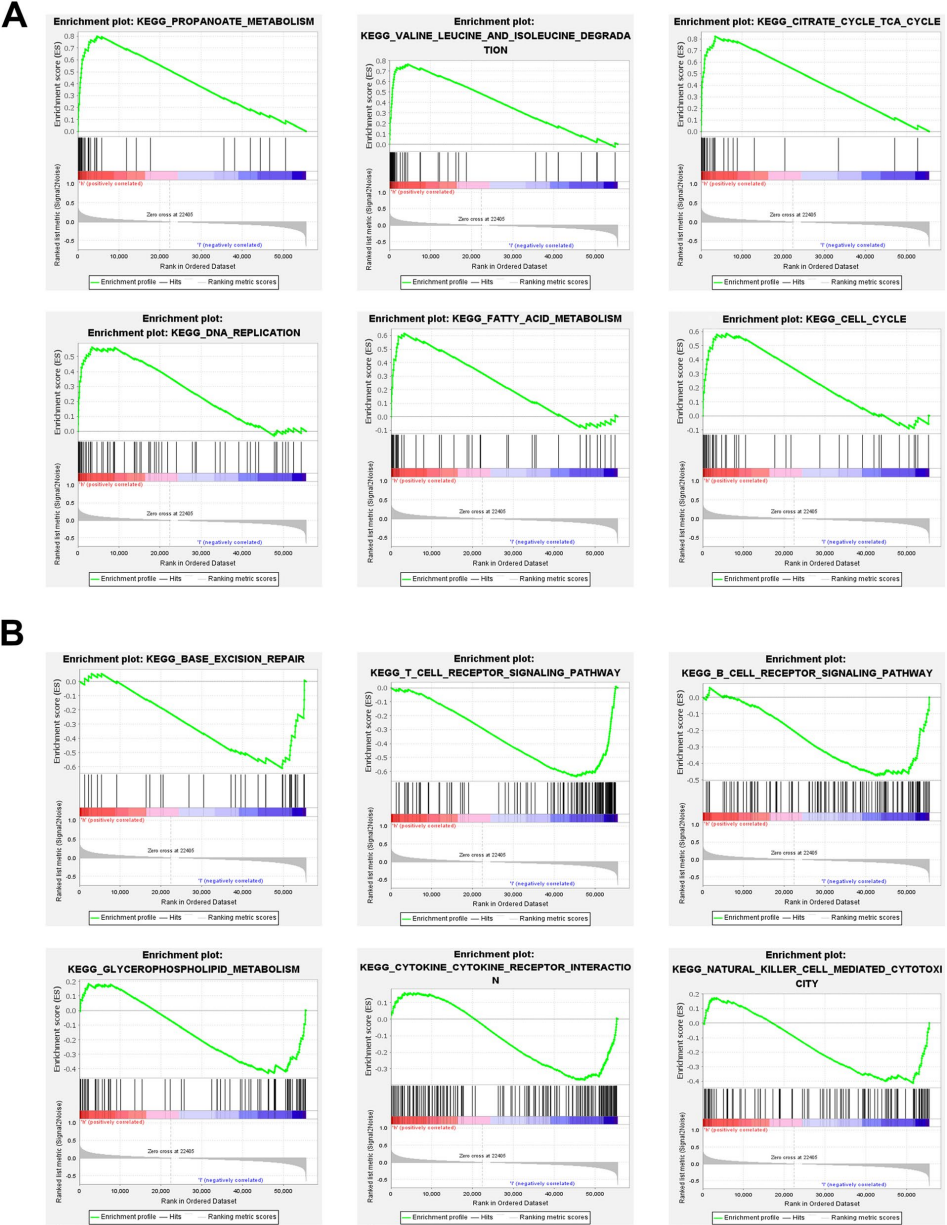
GSEA of high-risk and low-risk groups based on the FRLs prognostic signature. **A** GSEA results showing significant enrichment of metabolism pathways and cell proliferation pathways in the high-risk THCA patients. **B** GSEA results showing significant enrichment of immue-related pathways in the low-risk THCA patients

To explore the biological functions and signal transduction pathways associated with the FRLs, the differentially expressed genes between the high-risk and low-risk groups were used to perform Gene Set Enrichment Analysis (GSEA). The results showed that the metabolism pathways and cell proliferation pathways, such as propanoate metabolism, valine leucine and isoleucine degradation, citrate cycle tca cycle, DNA replication, fatty acid metabolism and cell cycle, were active in the high-risk THCA patients (Fig. 6A). While some immune-related pathways against cancer were significantly activated in the low-risk THCA patients, such as T cell receptor signaling pathway, natural killer cell-mediated cytotoxicity, B cell receptor signaling pathway and cytokine cytokine receptor interaction (Fig. 6B).

### Immune-Related pathways were activated in the FRLs model

**Fig. 7:**
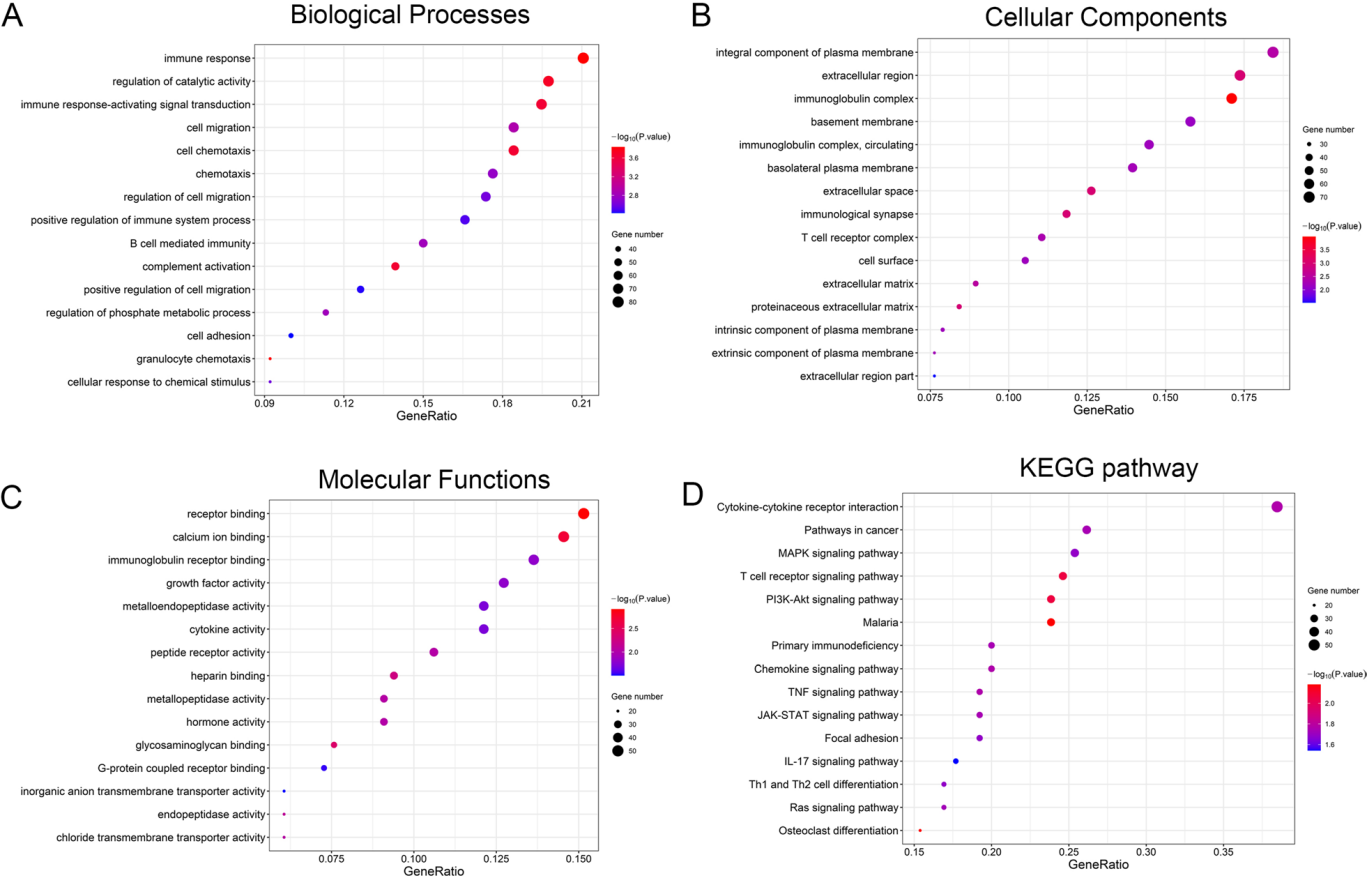
Results of GO and KEGG analyses. GO analysis showed that differentially expressed genes between high-risk and low-risk groups were obviously enriched in **A** immune-related biological processes, **B** immune-related cell components, **C** and immune-related molecular functions. **D** KEGG analysis showed that differentially expressed genes were enriched in immune-related pathways

In addition to GSEA, GO enrichment analysis and KEGG enrichment analysis were performed to determine the biological functions related to the FRLs. We used the aforementioned differentially expressed genes between the high-risk and low-risk groups for enrichment analysis and found that the differentially expressed genes were obviously enriched in many immune-related pathways, such as immune response, immune response-activating signal transduction, B cell-mediated immunity in biological processes (Fig. 7A), immunoglobulin complex, immunological synapse, T cell receptor complex in cellular components (Fig. 7B), immunoglobulin receptor binding, cytokine activity in molecular functions (Fig. 7C). The result of KEGG enrichment analysis also showed that the differentially expressed genes were enriched in cytokine-cytokine receptor interaction, T cell receptor signaling pathway, TNF signaling pathway and IL-17 signaling pathway (Fig. 7D).

### The immune cell infiltration landscape in THCA

**Fig. 8:**
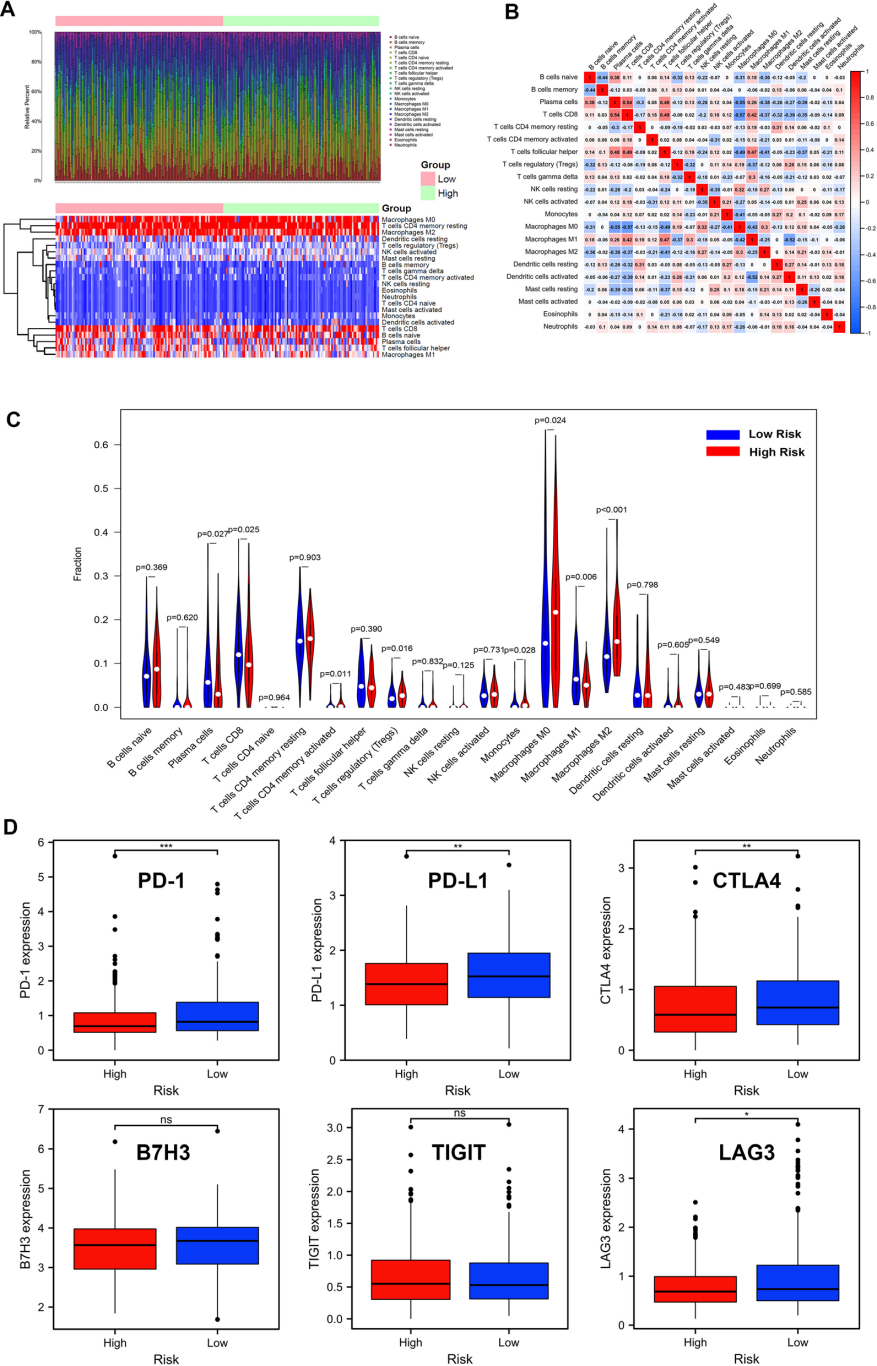
The immune cell infiltration landscape in THCA. **A** Barplot of the tumor-infiltrating cell proportions. **B** Heatmap of the tumor-infiltrating cell proportions. **C** Correlation matrix of immune cell proportions. **D** Violin plot showing the different proportions of tumor-infiltrating cells between high-risk group and low-risk groups. **E** The expression levels of immune checkpoint molecules in the high-risk group and low-risk group

The results of GSEA, GO enrichment analysis and KEGG enrichment analysis suggested that FRLs may be involved in immune-related functions in THCA. Therefore, we further explored the relationship between FRLs and anti-tumor immunity. CIBERSORT algorithm was used for investigating the immune cell infiltration landscape of the 502 THCA patients. The proportions of tumor-infiltrating immune cells were found to be significantly different between the high-risk group and the low-risk group (Fig. 8A). We also showed the correlation matrix of all tumor infiltrating immune cells (Fig. 8B). To compare the differences of infiltrating immune cells between the high-risk and low-risk groups, a violin plot was generated and showed that the proportions of T cells CD4 + memory activated (p = 0.011), T cells regulatory (Tregs) (p = 0.016), monocytes (p = 0.028), macrophages M0 (p = 0.0024) and macrophages M2 (p < 0.001) in the high-risk group were significantly higher than those in the low-risk group, while the proportions of plasma cells (p = 0.027), T cells CD8 (p = 0.025) and macrophages M1 (p = 0.006) in the high-risk group were lower than those in the low-risk group (Fig. 8C). Then we compared the expression levels of classic immune checkpoint molecules in the high-risk group and low-risk group, and found that some common immune checkpoint molecules such as PD-1, PD-L1, CTLA4 and LAG3 were all more abundantly expressed in the low-risk group than in the high-risk group. However, other immune checkpoint molecules, such as B7H3 and TIGHT, were not differentially expressed between the high-risk group and the low-risk group (Fig. 8D).

## Discussion

In this study, we systematically explored the relationship between lncRNAs and FRGs in THCA. The differentially expressed lncRNAs between THCA and normal tissues, FRLs, and prognostic lncRNAs were obtained from TCGA database and FerrDb database. Twenty-two prognostic differentially expressed FRLs were finally included for analyses. A novel prognostic model containing five FRLs was developed by further multivariate analysis. According to the prognostic model, we divided the patients of the training cohort, validation cohort and entire cohort into high-risk and low-risk groups. KM survival curves and time-dependent ROC curves between the high-risk group and the low-risk group were compared, and the differentially expressed genes between two groups were screened out. GSEA and functional enrichment analysis both showed that immune-related pathways were significantly differentially enriched between the two groups. Finally, we analyzed the infiltration level and the correlation matrix of all tumor-infiltrating immune cells in THCA. We also found that the expression levels of common immune checkpoint molecules in the low-risk group were higher than those in the high-risk group, which indicated that the low-risk group in THCA was immunologically “hot”.

Ferroptosis is an iron-dependent oxidative form of cell death associated with increased lipid peroxidation and insufficient capacity to eliminate lipid peroxides. Ferroptosis is distinct from other reported forms of cell death, namely apoptosis, necroptosis, and classic necrosis [28]. After several years of study, ferroptosis has been recognized as clinically important. Preliminary evidence suggests that ferroptosis suppresses tumor growth, progression and have potential benefits for cancer therapy in hepatocellular carcinoma, colorectal cancer, bladder cancer, lung cancer, thyroid cancer, pancreatic cancer, and prostate cancer [5]. For example, the E3 ligase MIB1 promotes proteasomal degradation of NRF2 and sensitizes lung cancer cells to ferroptosis [29]. Another study showed that miR-15a-3p regulated ferroptosis by targeting glutathione peroxidase GPX4 in colorectal cancer [30]. In addition, ferroptosis is also associated with exacerbation of other diseases, including infection, injury, and neurological degeneration. It has been reported that ferroptosis can exacerbate kidney injury, heart failure, bone marrow injury, brain injury, and spinal cord injury, and result in Huntington’s disease, rapid motor neuron degeneration, paralysis, Parkinson’s disease, stroke, and Alzheimer’s disease [31–33]. In these studies, many genes and small molecules have been shown to play important roles in the progression of ferroptosis. Zhou et al. built FerrDb that collects genes and small molecules and annotates them as regulators and markers of ferroptosis, also named as FRGs. We downloaded 241 FRGs from FerrDb as the basis of this study.

Many studies have recently found that lncRNAs can regulate the progression of various tumors by affecting ferroptosis. For example, lncRNA RP11-89 facilitates tumorigenesis and ferroptosis resistance through PROM2-activated iron export by sponging miR-129-5p in bladder cancer [34]. lncRNA LINC00336 inhibits ferroptosis in lung cancer by functioning as a competing endogenous RNA [35]. In addition, some studies constructed the FRGs signature to predict prognosis of several cancers, such gliomas, gastric cancer and lung adenocarcinoma [36–38]. However, prognostic models based on FRLs in THCA are still limited. Therefore, we performed Pearson correlation analysis between the discovered FRGs and lncRNAs to identify FRLs. By analyzing the intersections between differentially expressed lncRNAs between tumor and normal tissues, and prognosis-related lncRNAs, 22 FRLs were identified in THCA, which were named as prognostic differentially expressed FRLs. Furthermore, five FRLs (AC055720.2, DPP4-DT, AC012038.2, LINC02454 and LINC00900) were selected to construct a prognostic signature based on their performance in the multivariate cox regression analysis. According to the five FRLs prognostic signature, we divided the training cohort, validation cohort, and entire cohort into high-risk and low-risk groups. Notably, we found that the OS of patients in the high-risk group was significantly shorter than that in the low-risk group. Furthermore, the ROC curve showed that the AUC values of the FRLs prognostic signature in the training cohort, validation cohort, and entire cohort were higher than those of other traditional prognostic factors.

Growing evidence has suggested that immune cells in TME play vital roles in tumorigenesis. These innate immune cells, including macrophages, neutrophils, dendritic cells, innate lymphoid cells, myeloid-derived suppressor cells, and natural killer cells, potentially possess tumour-inhibiting or tumour-promoting functions [39]. THCA is considered as the “inflammatory tumor” and cancer-related inflammation could be the potential diagnostic and therapeutic target in THCA patients [40]. Ferroptosis also plays an important immunological role in the process of tumour surveillance by affecting tumour immunity [39, 41]. For example, CD8 + T cells suppress tumor development by promoting tumor ferroptosis (31043744). CD36-mediated ferroptosis dampens the effector function of intratumoral CD8 + T cells and decreases their antitumor ability [42]. However, the role of ferroptosis, especially of the FRLs, in THCA immune microenvironment is still unclear. In our study, through GSEA and functional enrichment analysis, immune-related pathways, including T cell receptor signaling pathway, natural killer cell-mediated cytotoxicity, B cell receptor signaling pathway and cytokine cytokine receptor interaction were found to be activated and inhibited in the high-risk and low-risk groups, respectively. Therefore, FRLs were proposed to be closely related to anti-tumor immunity in THCA. Subsequently, we further analyzed the relationship between FRLs and immune cell infiltration in THCA. CIBERSORT algorithm was used to calculate the relative abundance of different types of tumor-infiltrating immune cells. Compared with the low-risk group, the proportions of infiltrating tumor-killing immune cells, such as plasma cells, CD8 + T cells and M1 macrophages, in the THCA tissues of the high-risk group were significantly reduced, whereas those of infiltrating tumor-promoting immune cells, such as M2 macrophages and Tregs, were significantly increased [43, 44]. Therefore, ferroptosis was concluded to significantly correlate with the activity of tumor-infiltrating immune cells in THCA.

In addition, immune checkpoint molecules, including PD-1, PD-L1, CTLA4, and LAG3, were revealed to be more remarkably expressed in the low-risk group. Our study suggested that the low-risk score group is likely to present an immunogenic TME. We inferred that THCA patients with low-risk scores might respond better to immune checkpoint blockage therapy, which could also account for the promising survival outcome in this group.

Nevertheless, there were some limitations in our study. The FRLs prognostic model was only constructed and verified using data from TCGA public database. The universality and reliability of the prognostic model remain to be further verified in an external prospective, multi-center, real-world cohort. In addition, although our study revealed the relationship between FRLs and anti-tumor immunity, the underlying mechanisms need to be further explored by experiments.

## Conclusion

In summary, our study used the TCGA THCA dataset to construct a novel FRLs prognostic model which could precisely predict the prognosis of THCA patients. These FRLs potentially mediate anti-tumor immunity and serve as therapeutic targets for THCA, which provided the novel insight into treatment of THCA.

## Supplementary Information

**Additional file 1: Table S1**. Ferroptosis-related genes.

**Additional file 2: Table S2**. Akaike information criterion for the prognostic signature.

**Additional file 3: Table S3**. Primer sequences used for qRT-PCR.

## Data Availability

Publicly available datasets were analyzed in this study. This data can be found here: The data of this study were downloaded from TCGA database (https://portal.gdc.cancer.gov/repository).
