# Supplementary material for: Construction of a ferroptosis-related five-lncRNA signature for predicting prognosis and immune response in thyroid carcinoma
Source: Cancer Cell Int. 2022 Sep 29;22:296. doi: 10.1186/s12935-022-02674-z (PMC9520852; doi:10.1186/s12935-022-02674-z)
Supplement: Supplementary file 2 — Additional file 2: Table S2. Akaike information criterion for the prognostic signature. [file 12935_2022_2674_MOESM2_ESM.docx]

**Table 2 Akaike information criterion for the prognostic signature.**

| **Model** | **Prognostic signature combination** | **AIC** |
| --- | --- | --- |
| 1 | DOCK9-DT+AC046143.1+AC022509.2+MIR181A2HG+AF131215.7+AC055720.2+AC084375.1+LINC02471+DPP4-DT+AL162511.1+HMGA2-AS1+AL031985.3+AC141930.1+AC012038.2+TBILA+AL158206.1+FAM111A-DT+LINC02454+AC254633.1+AC005479.2+AC007255.1+LINC00900 | 184.02 |
| 2 | DOCK9-DT+AC046143.1+AC022509.2+AF131215.7+AC055720.2+AC084375.1+LINC02471+DPP4-DT+AL162511.1+HMGA2-AS1+AL031985.3+AC141930.1+AC012038.2+TBILA+AL158206.1+FAM111A-DT+LINC02454+AC254633.1+AC005479.2+AC007255.1+LINC00900 | 182.04 |
| 3 | DOCK9-DT+AC046143.1+AC022509.2+AF131215.7+AC055720.2+AC084375.1+LINC02471+DPP4-DT+AL162511.1+HMGA2-AS1+AL031985.3+AC141930.1+AC012038.2+TBILA+AL158206.1+FAM111A-DT+LINC02454+AC254633.1+AC005479.2+LINC00900 | 180.06 |
| 4 | DOCK9-DT+AC046143.1+AC022509.2+AC055720.2+AC084375.1+LINC02471+DPP4-DT+AL162511.1+HMGA2-AS1+AL031985.3+AC141930.1+AC012038.2+TBILA+AL158206.1+FAM111A-DT+LINC02454+AC254633.1+AC005479.2+LINC00900 | 178.09 |
| 5 | DOCK9-DT+AC046143.1+AC022509.2+AC055720.2+AC084375.1+LINC02471+DPP4-DT+AL162511.1+HMGA2-AS1+AL031985.3+AC141930.1+AC012038.2+TBILA+AL158206.1+FAM111A-DT+LINC02454+AC005479.2+LINC00900 | 176.14 |
| 6 | DOCK9-DT+AC046143.1+AC022509.2+AC055720.2+AC084375.1+LINC02471+DPP4-DT+AL162511.1+HMGA2-AS1+AL031985.3+AC141930.1+AC012038.2+TBILA+AL158206.1+FAM111A-DT+LINC02454+LINC00900 | 174.23 |
| 7 | DOCK9-DT+AC046143.1+AC022509.2+AC055720.2+AC084375.1+LINC02471+DPP4-DT+AL162511.1+HMGA2-AS1+AL031985.3+AC141930.1+AC012038.2+AL158206.1+FAM111A-DT+LINC02454+LINC00900 | 172.42 |
| 8 | AC046143.1+AC022509.2+AC055720.2+AC084375.1+LINC02471+DPP4-DT+AL162511.1+HMGA2-AS1+AL031985.3+AC141930.1+AC012038.2+AL158206.1+FAM111A-DT+LINC02454+LINC00900 | 170.59 |
| 9 | AC046143.1+AC022509.2+AC055720.2+AC084375.1+LINC02471+DPP4-DT+AL162511.1+HMGA2-AS1+AC141930.1+AC012038.2+AL158206.1+FAM111A-DT+LINC02454+LINC00900 | 168.72 |
| 10 | AC046143.1+AC022509.2+AC055720.2+AC084375.1+LINC02471+DPP4-DT+AL162511.1+HMGA2-AS1+AC012038.2+AL158206.1+FAM111A-DT+LINC02454+LINC00900 | 166.94 |
| 11 | AC046143.1+AC022509.2+AC055720.2+AC084375.1+LINC02471+DPP4-DT+HMGA2-AS1+AC012038.2+AL158206.1+FAM111A-DT+LINC02454+LINC00900 | 165.18 |
| 12 | AC022509.2+AC055720.2+AC084375.1+LINC02471+DPP4-DT+HMGA2-AS1+AC012038.2+AL158206.1+FAM111A-DT+LINC02454+LINC00900 | 163.54 |
| 13 | AC055720.2+AC084375.1+LINC02471+DPP4-DT+HMGA2-AS1+AC012038.2+AL158206.1+FAM111A-DT+LINC02454+LINC00900 | 161.82 |
| 14 | AC055720.2+AC084375.1+DPP4-DT+HMGA2-AS1+AC012038.2+AL158206.1+FAM111A-DT+LINC02454+LINC00900 | 160.32 |
| 15 | AC055720.2+AC084375.1+DPP4-DT+AC012038.2+AL158206.1+FAM111A-DT+LINC02454+LINC00900 | 159.26 |
| 16 | AC055720.2+DPP4-DT+AC012038.2+AL158206.1+FAM111A-DT+LINC02454+LINC00900 | 158.72 |
| 17 | AC055720.2+DPP4-DT+AC012038.2+FAM111A-DT+LINC02454+LINC00900 | 158.05 |
| 18 | AC055720.2+DPP4-DT+AC012038.2+LINC02454+LINC00900 | 158 |
