# Supplementary material for: Construction of a ferroptosis-related five-lncRNA signature for predicting prognosis and immune response in thyroid carcinoma
Source: Cancer Cell Int. 2022 Sep 29;22:296. doi: 10.1186/s12935-022-02674-z (PMC9520852; doi:10.1186/s12935-022-02674-z)
Supplement: Supplementary file 3 — Additional file 3: Table S3. Primer sequences used for qRT-PCR. [file 12935_2022_2674_MOESM3_ESM.docx]

**Table S3. Primer sequences used for qRT-PCR.**

| **Primer** | **Sense (5′–3′)** | **Antisense (5′–3′)** |
| --- | --- | --- |
| LINC00900 | AACGCTGACACTGATGACCC | TTGAGGGGAGGGGTGAAAGA |
| LINC02454 | GCTTGAACATCGTCCTCCTC | TGTTCTCTGTGGGAATGCAA |
| DPP4-DT | CTCTCCTCCTCCCTCTTCCAACTG | GCACAGGCTTAACTCTCCAGACTTC |
| GAPDH | GCACCGTCAAGGCTGAGAAC | TGGTGAAGACGCCAGTGGA |
